# Duration and accuracy of automated stroke CT workflow with AI-supported intracranial large vessel occlusion detection

**DOI:** 10.1038/s41598-023-39831-x

**Published:** 2023-08-02

**Authors:** Sander E. Temmen, Marinus J. Becks, Steven Schalekamp, Kicky G. van Leeuwen, Frederick J. A. Meijer

**Affiliations:** Department of Medical Imaging, Radboud University Medical Center, Geert Grooteplein Zuid 10, 766, PO Box 9101, 6500HB Nijmegen, The Netherlands

**Keywords:** Brain imaging, Computed tomography

## Abstract

The Automation Platform (AP) is a software platform to support the workflow of radiologists and includes a stroke CT package with integrated artificial intelligence (AI) based tools. The aim of this study was to evaluate the diagnostic performance of the AP for the detection of intracranial large vessel occlusions (LVO) on conventional CT angiography (CTA), and the duration of CT processing in a cohort of acute stroke patients. The diagnostic performance for intracranial LVO detection on CTA by the AP was evaluated in a retrospective cohort of 100 acute stroke patients and compared to the diagnostic performance of five radiologists with different levels of experience. The reference standard was set by an independent neuroradiologist, with access to the readings of the different radiologists, clinical data, and follow-up. The data processing time of the AP for ICH detection on non-contrast CT, LVO detection on CTA, and the processing of CTP maps was assessed in a subset 60 patients of the retrospective cohort. This was compared to 13 radiologists, who were prospectively timed for the processing and reading of 21 stroke CTs. The AP showed shorter processing time of CTA (mean 60 versus 395 s) and CTP (mean 196 versus 243–349 s) as compared to radiologists, but showed lower sensitivity for LVO detection (sensitivity 77% of the AP vs mean sensitivity 87% of radiologists). If the AP would have been used as a stand-alone system, 1 ICA occlusion, 2 M1 occlusions and 8 M2 occlusions would have been missed, which would be eligible for mechanical thrombectomy. In conclusion, the AP showed shorter processing time of CTA and CTP as compared with radiologists, which illustrates the potential of the AP to speed-up the diagnostic work-up. However, its performance for LVO detection was lower as compared with radiologists, especially for M2 vessel occlusions.

## Introduction

In the treatment of stroke, a quick and accurate diagnostic workup is crucial to select patients for therapy, aimed to limit brain tissue damage and to improve long-term outcomes^1^. The implementation of an automated CT workflow with AI-aided diagnosis has the potential to enhance and expedite this process^2,3^. The Automation Platform (AP) (Canon Medical Systems) is a software platform designed to support the radiologists’ workflow and includes AI-based tools. The stroke CT package embedded in the AP provides diagnostic support and can speed up the diagnostic imaging process by automatically processing brain CT perfusion (CTP) and highlighting potential abnormalities on non-contrast CT (NCCT), or conventional CT angiography (CTA) and by providing an overview of relevant imaging findings.

The aim of this study was to assess the diagnostic performance of the AP for the detection of intracranial large vessel occlusions (LVO) on conventional CTA, as well as the duration of CT processing, in a cohort of acute stroke patients.

## Materials and methods

### Study populations

We used data from a retrospective cohort of 100 stroke patients from a previous study to evaluate the diagnostic performance for LVO detection on CTA for both the AP and radiologists, as well as the duration of data processing of the AP^4^. Inclusion criteria in this cohort were age > 18 years and onset of symptoms of less than 9 h. Exclusion criteria were intracranial hemorrhage, another diagnosis explaining the symptoms, and previous (endovascular) brain surgery. The characteristics of the study population are provided in Table 1.

**Table 1:** Patient characteristics for diagnostic performance evaluation of large vessel occlusion.

Patient characteristics	N = 100
Male gender (%)	57 (57%)
Age Y, mean (SD)	64 (13.17)
NIHSS mean (SD)	6.28 (6.55)
mRs mean (SD)	2.72 (1.59)
Side of neurological symptoms	
Left (%)	35 (35%)
Right (%)	56 (56%)
Indifferent (%)	9 (9%)
LVO (%)	23 (23%)
ICA (%)	3 (13%)
MCA M1 (%)	10 (43%)
MCA M2 (%)	10 (43%)
Other vessel occlusions	29 (29%)
MCA M3 (%)	9 (31%)
Basilar artery (%)	2 (6.9%)
Vertebral artery (%)	4 (13.7%)
PCA 1 (%)	5 (17%)
PCA 2 (%)	2 (6.9%)
PCA 3 (%)	2 (6.9%)
ACA 2 (%)	1 (3.4%)
AICA/PICA (%)	3 (10%)
SCA (%)	1 (3.4%)
No occlusions (%)	48 (48%)

NIHSS: National Institute of Health Stroke Scale; mRs: Modified Rankin Score; SD: standard deviation; ICA: internal carotid artery; MCA: middle cerebral artery; PCA: posterior cerebral artery; ACA: anterior cerebral artery; AICA/PICA: anterior inferior cerebellar artery/posterior inferior cerebellar artery; SCA: superior cerebellar artery.

We measured the reading time of NCCT and CTA and the duration of CTP processing by 13 radiologists without the use of the AP in a separate, prospective cohort of 21 patients who presented with stroke-like symptoms.

All patients of the two study cohorts received the same stroke CT scanning protocol, including non-contrast brain CT, head-neck CTA and whole-brain CTP on a 320-row detector CT scanner (Aquilion One, Vision or Genesis edition, Canon Medical Systems).

The study was approved by the medical-ethical committee, and informed consent was waived because of the retrospective collection of the study data (IRB Oost-Nederland, Radboudumc, Nijmegen, The Netherlands). The study was performed in accordance with relevant guidelines and regulations. Clinical data and results of imaging analyses were anonymized and stored in a protected database Castor EDC (http://www.castoredc.com), following Good Clinical Practice (GCP) and European Data Protection Directive guidelines.

### The Automation Platform Stroke CT package

The Stroke CT package implemented in the Automation Platform (base version 1.3, Canon Medical Systems, Otawara, Japan) was evaluated. The embedded AI algorithm to detect LVOs is an integrated third party application called CINA LVO (version 1.0.3, Avicenna.ai) and its intended use is to detect distal internal carotid artery (ICA), M1 occlusions and proximal M2 occlusions. NCCTs were analyzed with CINA intracerebral hemorrhage (ICH) application (version 1.0.4, Avicenna.ai) also part of the Stroke CT package. The conventional and AP supported workflows are illustrated in the supplementary figure.

### Assessment of large vessel occlusion detection

An LVO is defined as an occlusion of the distal internal carotid artery (ICA) and M1 or M2 segments of the middle cerebral artery (MCA) eligible for mechanical thrombectomy^4^. The CTAs were retrospectively analyzed on a PACS system (Impax 6.6.1.0 2015, Agfa Healthcare, Morsel, Belgium) for the presence or absence of a LVO by two neuroradiologists, one radiologist and two radiology residents. The readers were only provided the sidedness of the neurological deficit without further details and they did not have access to non-contrast CT or perfusion CT. The AP evaluated the same set of CTAs.

The reference standard was set by an independent neuroradiologist, with access to the results of the different radiologists, clinical data, and follow-up. Measures of diagnostic performance were calculated. A p-value below 0.05 was considered statistically significant.

### Assessment of the processing time

The duration of automated processing of NCCT, CTA and CTP for the AP was timed in 60 cases of the retrospective cohort. The duration of the processing and reading of 13 different radiologists (4 neuroradiologists, 4 non-neuroradiologists and 5 radiology residents) in clinical practice was timed in 21 prospective stroke CTs. Out of the 21 scans, 8 were read by a neuroradiologist, 4 by a non-neuroradiologist and 9 by a radiology resident. An independent t-test was used to compare the four groups (AP, neuroradiologists, non-neuroradiologists and radiology residents) for the duration of processing the CTP. A p-value < 0.05 was considered statistically significant. Furthermore, the minimum and maximum duration of the different measurements were provided.

## Results

### Diagnostic performance for LVO detection

The AP correctly detected 2 out of 3 distal ICA occlusions, 8 out of 10 M1 occlusions and 2 out of 10 M2 occlusions. Sensitivity for LVO detection for the AP was 77% (95%-CI [46–95%]) for distal ACI and M1 occlusions, and 52% (95%-CI [30–73%]) when M2 vessel occlusions were included in the analysis. Specificity was 99% (95%-CI [93–100%]) for overall LVO detection. Radiologists’ mean sensitivity for LVO detection was 87% and ranged between 78 and 91%. They had a similar high specificity (mean 98%) compared to the AP. The exclusion of M2 occlusions had a minor effect on sensitivity (86%) or specificity (97%) for radiologists. When all LVO’s were considered, the diagnostic performance for the AP for LVO detection was therefore substantially lower as compared to radiologists. The complete results are presented in Tables 2 and 3.

**Table 2:** Diagnostic performance of the five radiologists and the Automation Platform for ICA and M1 vessel occlusion detection.

ICA + M1 MCA	Sensitivity % (95%-CI)	Specificity % (95%-CI)	PPV % (95%-CI)	NPV % (95%-CI)	Accuracy % (95%-CI)	ROC (area under the curve)
Observer 1 (Neuroradiologist)	92 (64–100)	97 (91–100)	100	99 (92–100)	99 (94–100)	0.96
Observer 2 (General radiologist)	92 (64–100)	97 (91–100)	86 (60–96)	99 (92–100)	97 (91–99)	0.95
Observer 3 (Radiology resident)	85 (55–98)	97 (91–100)	85 (58–96)	97 (91–99)	96 (89–99)	0.91
Observer 4 (Radiology resident)	77 (49–95)	99 (93–100)	91 (58–99)	96 (90–99)	96 (89–99)	0.88
Observer 5 (Neuroradiologist)	85 (55–98)	97 (91–100)	85 (58–96)	97 (91–99)	96 (89–99)	0.91
Automation Platform	77 (46–95)	99 (93–100)	91 (58–99)	96 (90–99)	96 (89–99)	0.88

ICA: internal carotid artery; MCA: middle cerebral artery; PPV: positive predictive value; NPV: negative predictive value; ROC: receiver-operating characteristic.

**Table 3:** Diagnostic performance of the five radiologists and the Automation Platform for overall LVO detection (ICA, M1, M2).

All anterior LVOs (distal ICA, M1 and M2)	Sensitivity % (95%-CI)	Specificity % (95%-CI)	PPV % (95%-CI)	NPV % (95%-CI)	Accuracy % (95%-CI)	ROC (area under the curve)
Observer 1 (Neuroradiologist)	91 (72–99)	100 (95–100)	100	97 (91–99)	98 (93–100)	0.96
Observer 2 (General radiologist)	83 (61–95)	97 (91–100)	90 (70–97)	95 (88–98)	94 (87–98)	0.90
Observer 3 (Radiology resident)	91 (72–99)	97 (91–100)	91 (73–98)	91 (73–98)	96 (90–99)	0.94
Observer 4 (Radiology resident)	78 (56–93)	99 (93–100)	95 (72–99)	94 (87–97)	94 (87–98)	0.89
Observer 5 (Neuroradiologist)	91 (72–99)	97 (91–100)	91 (73–98)	91 (73–98)	96 (90–99)	0.94
Automation Platform	52 (30–73)	99 (93–100)	92 (62–99)	87 (82–91)	88 (80–94)	0.75

LVO: large vessel occlusion; PPV: positive predictive value; NPV: negative predictive value; ROC: receiver-operating characteristic.

### Duration of CT readings by AP and radiologists

**Figure 1:**
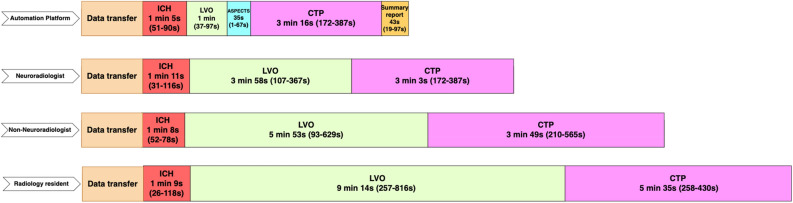
Timelines of the duration of data processing for the Automation Platform and radiologists with different levels of experience (4 neuroradiologists, 4 non-neuroradiologists and 5 radiology residents). Each timeframe represents the mean duration of processing/reading, with the minimum and maximum times (range) provided in seconds. The durations were times manually. ICH: intracerebral hemorrhage, LVO: large vessel occlusion, ASPECTS: Alberta Stroke Program Early CT Score, CTP: CT perfusion.

The duration of processing for the AP and three groups of radiologists are presented in Fig. 1. The evaluation time for intracranial hemorrhage detection on NCCT was comparable between the AP (mean: 65 s) and radiologists (mean: 69 s). The AP needed on average 60 s for the assessment of CTA reading for LVO detection, which was shorter compared to the groups of radiologists (mean: 395 s). Mean duration of CTP processing by the AP was 196 s, which was significantly shorter than non-neuroradiologists (mean: 349 s, p = 0.01) and radiology residents (mean 335 s, p < 0.001).

## Discussion

Our study found that while the AP had a lower performance than radiologists in detecting LVOs on CTA, it was able to process CTP and detect LVOs in a shorter time than radiologists, highlighting the potential of automated workflow support in speeding up the diagnostic work, particularly for less experienced radiologists. If the AP would have been used as a stand-alone system, 1 ICA occlusion, 2 M1 occlusions and 8 M2 occlusions would have been missed, which would be eligible for mechanical thrombectomy. The low detection rate for M2 vessel occlusions is in line with previous studies showing low sensitivity for M2 vessel occlusion detection by the same and other AI-algorithms^4–8^. Most AI algorithms currently available are only intended for M1 and ICA occlusion detection, so it is important to ensure that end-users are aware of the intended purpose of the AI algorithm. In clinical practice, an AI-tool could especially add value by detecting smaller but treatable vessel occlusions, such as M2 vessel occlusions, which may be overlooked by radiologists. For now, the potential to improve healthcare outcomes and to save costs by using this AI-tool to reduce the number of missed LVOs, as discussed in an early health technology assessment study^2^, would probably not yet be realized. It should be noted that the availability of more detailed clinical information, non-contrast CT and the use of CTP maps helps to improve the detection of intracranial vessel occlusions^9,10^.

Although the duration of NCCT evaluation for ICH detection was similar between AP and radiologists, the actual performance of ICH detection was not evaluated in our study as patients with intracranial hemorrhage were excluded from our cohort. A clear advantage of the AP was the automated processing of CTP maps, which saves time for radiologists, especially for those who were less experienced.

Our study has some limitations. The study group is rather small and stroke mimickers were excluded from the dataset, which probably resulted in a higher than expected incidence of LVOs as compared to daily clinical practice. It is however not expected that this would have a significant influence on the results of our study, because LVOs are not expected to be present in stroke mimickers. A more diverse and larger population size would be required to comprehensively evaluate the AP's performance. Additionally, the AP was evaluated as a stand-alone system, and its actual (added) value for radiologists in LVO detection was not assessed. It remains to be determined whether the diagnostic performance and confidence of less experienced readers actually improves by the use of an AI-tool. Incorrect results of the AI–based tool could impair the performance of radiologists with varying levels of expertise, which is called automation bias^11^. Furthermore, the duration of the AP for LVO and ICH assessment and CTP processing was retrospectively compared with the prospective timing of radiologists, as the AP was not yet implemented in clinical practice, which limited a direct comparison between the AP and radiologists. It should also be emphasized that the AP cannot be used as a stand-alone system, because of the moderate diagnostic performance but also because of its narrow target-use, and that radiologists should verify the AP's results, which takes time and results in a smaller time difference between the AP and radiologists. Finally, the CTP datasets' slice thickness was 0.5 mm, and data was sent to the AP in a non-enhanced DICOM format. Changing the slice thickness to 1.0 mm and transferring data in an enhanced DICOM format could decrease the duration of data transfer and processing in clinical practice.

## Conclusion

The AP showed shorter processing time of CTA and CTP as compared with radiologists, which illustrates the potential of the AP to speed-up the diagnostic work-up. However, its performance for LVO detection was lower as compared with radiologists, especially for M2 vessel occlusions.

## Supplementary Information

Supplementary Figure 1.

Supplementary Table 1.

## Data Availability

All data generated and analyzed during this study are included in this published article and its supplementary information files.
