# Supplementary material for: Duration and accuracy of automated stroke CT workflow with AI-supported intracranial large vessel occlusion detection
Source: Sci Rep. 2023 Aug 2;13:12551. doi: 10.1038/s41598-023-39831-x (PMC10397283; doi:10.1038/s41598-023-39831-x)
Supplement: Supplementary file 1 — Supplementary Figure 1. [file 41598_2023_39831_MOESM1_ESM.docx]

**Supplementary Figure 1**


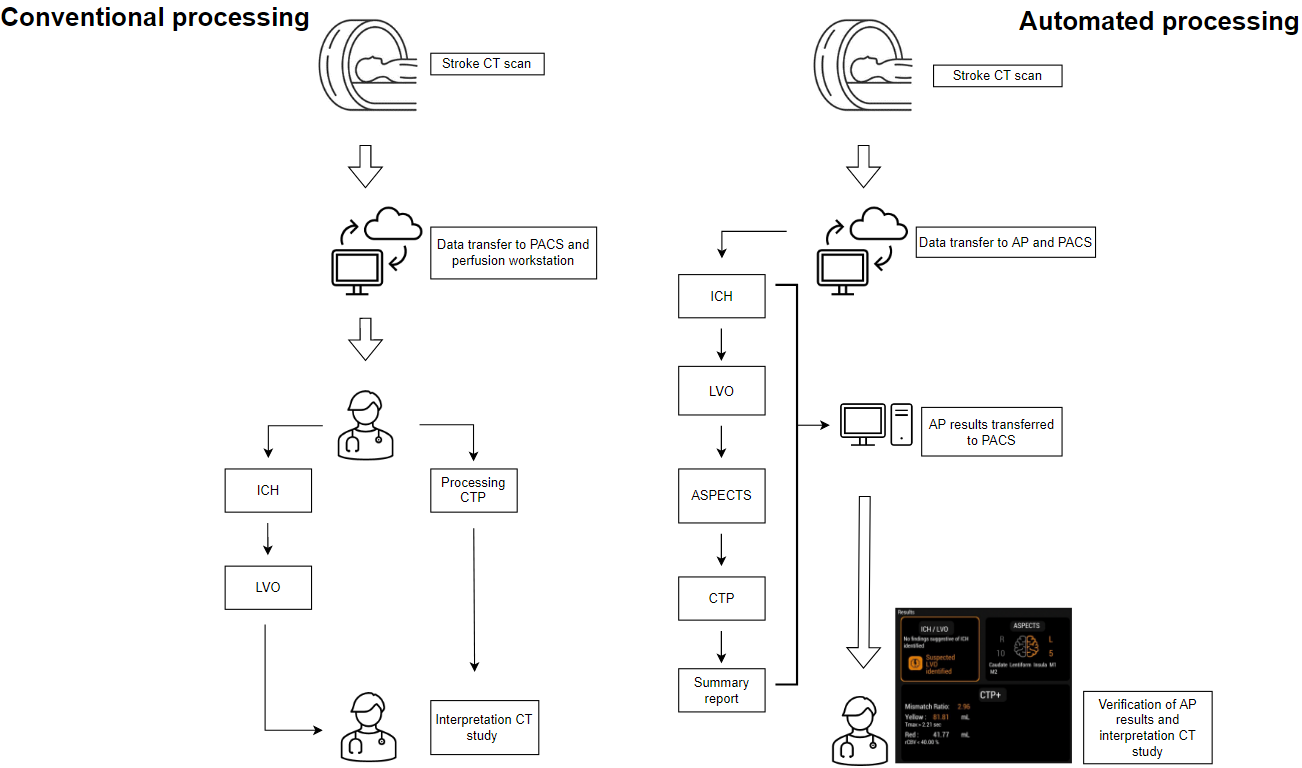


**Figure** Visualization of the conventional vs AI-supported workflow.
Abbreviations: PACS: Picture Archiving and Communication System, ICH: Intracerebral haemorrhage, CTP: CT Perfusion, LVO: Large vessel occlusion, AP: Automation Platform, ASPECTS: Alberta Stroke Program Early CT score*.*
