# Supplementary material for: Duration and accuracy of automated stroke CT workflow with AI-supported intracranial large vessel occlusion detection
Source: Sci Rep. 2023 Aug 2;13:12551. doi: 10.1038/s41598-023-39831-x (PMC10397283; doi:10.1038/s41598-023-39831-x)
Supplement: Supplementary file 2 — Supplementary Table 1. [file 41598_2023_39831_MOESM2_ESM.docx]

**Supplementary Table 1**

*Duration of manual and automated processing. The mean duration is per corresponding component given in seconds and the minimum and maximum times (range) are provided.*

| **Neuroradiologist**    ICH  LVO  CTP | **Mean (range)**  71s (31-116s)  238s (107-367s)  243s (172-387s) |
| --- | --- |
| **Non-neuroradiologist**    ICH  LVO  CTP | 68s (52-78s)  353s (93-629s)  349s (210-565s) |
| **Radiology resident**    ICH  LVO  CTP | 69s (26-118s)  554s (257-816s)  335s (258-430s) |
| **Automation Platform**  ICH LVO ASPECTS CTP  Summary report | 65s (51-90s)  60s (37-97s)  35s (1-67s)  196s (145-255s)  43s (19-97s) |

*Abbreviations: ICH: Intracerebral hemorrhage, LVO: Large vessel occlusion, ASPECTS: Alberta Stroke Program Early CT Score, CTP: CT Perfusion.*
